# Potential Functional Snacks: Date Fruit Bars Supplemented by Different Species of *Lactobacillus* spp.

**DOI:** 10.3390/foods10081760

**Published:** 2021-07-29

**Authors:** Maria Maisto, Giuseppe Annunziata, Elisabetta Schiano, Vincenzo Piccolo, Fortuna Iannuzzo, Rosaria Santangelo, Roberto Ciampaglia, Gian Carlo Tenore, Ettore Novellino, Paolo Grieco

**Affiliations:** 1 Department of Pharmacy, University of Naples Federico II, Via Domenico Montesano 59, 80131 Naples, Italy; giuseppe.annunziata@unina.it (G.A.); elisabetta.schiano@unina.it (E.S.); vincenzo.piccolo3@unina.it (V.P.); fortuna.iannuzzo@unina.it (F.I.); roberto.ciampaglia@unina.it (R.C.); giancarlo.tenore@unina.it (G.C.T.); paolo.grieco@unina.it (P.G.); 2 Pharma Biomateck s.r.l., Frattamaggiore, 80027 Naples, Italy; santangelo@pharmabiomateck.it; 3 NGN Healthcare—New Generation Nutraceuticals s.r.l., Torrette Via Nazionale 207, 83013 Mercogliano, Italy; ngnhealthcare@gmail.com

**Keywords:** lactofermentation, probiotic, date fruit bars, functional snack, polyphenols

## Abstract

The influence of the addition of four different potential probiotic strains, *Lactiplantibacillus plantarum* subsp. *plantarum* (*L. plantarum*), *Lactobacillus delbruekii* subsp. *bulgaricus* (*L. bulgaricus*), *Lactobacillus acidophilus* (*L. acidophilus*) and *Lactinocaseibacillus rhamnosus* (*L. rhamnosus*), in date fruit-based products was investigated in order to evaluate the possibility of producing a functional snack. All bacterial strains tested were able to grow in date fruit palp, reaching probiotic concentrations ranging from 3.1 × 10^9^ to 4.9 × 10^9^ colony-forming units after 48 h of fermentation, and the pH was reduced to 3.5–3.7 or below. The viability of inoculated probiotic bacteria after 4 weeks of storage at 4 °C was slightly reduced. Some biochemical features of the fermented snacks, such as the total phenolic content (TPC), antioxidant activity and detailed polyphenolic profile, were also evaluated. After fermentation, changes in the polyphenol profile in terms of increased free phenolic compounds and related activity were observed. These results may be attributed to the enzymatic activity of *Lactobacillus* spp. in catalyzing both the release of bioactive components from the food matrix and the remodeling of polyphenolic composition in favor of more bioaccessible molecules. These positive effects were more evident when the snack were fermented with *L. rhamnosus*. Our results suggest the use of lactic acid fermentation as an approach to enhance the nutritional value of functional foods, resulting in the enhancement of their health-promoting potential.

## 1. Introduction

Palm date fruit is one of the oldest fruits consumed by man. It is well known in folklore beliefs that palm date fruit possesses extraordinary health-promoting effects. In ancient times, it was largely used for its extraordinary effects on fertility and sexual performance, to care for gastrointestinal disturbances, but also to treat respiratory disease such as bronchitis and asthma [1,2]. Today, the latter beneficial effects have been scientifically studied and documented, and pre-clinical and clinical studies have confirmed the wide latter spectrum of health benefits after treatment with palm date fruit extract [3].

From the chemical point of view, the strength of this fruit is not only its peculiar polyphenolic content, characterized mainly in phenolic acid, followed by flavonoids, procyanidins, carotenoids, and sterols [3], but also its relevant nutritional properties, and especially its energy boosters. Particularly, palm date fruits are a rich source of minerals, such as potassium (864 mg/100 g), calcium (70.7 mg/100 g), sodium (32.9 mg/100 g), iron (0.3–6.03 mg/100 g), zinc (0.5 mg/100 g) and magnesium (64.2 mg/100 g), that are vital for human physiological process such as respiration (Na+), performance of the immune response (Zn) and physical potency (Fe) [4]. Palm date fruits are also a precious fruit, especially for their fiber content, mainly insoluble fiber (11.5 g/100 g) [5], protein content (2.5–6.5 g/100 g) [6], and of essential amino acids such as arginine and histidine that are fundamental for human health [7,8,9]. Moreover, the high level of glucose and fructose, easily absorbable at the intestinal level, make this fruit one of the most ancient and diffuse energy sources.

Over the last few decades, western nutraceutical and food industries have placed an increasing interest in the formulation of fruit- or vegetable-based fermented foods. These products have found a rapid diffusion on the nutraceutical and food market due to several reasons, primarily nutrition–health approaches, food safety, advantageous sensorial changing, shelf-life prolongation, the facility of preparation, the valorization of unused raw vegetal material, and sustainable development [10,11,12].

The main microorganism species employed for the formulation of such products are bacteria (manly *Bacillus subtilis*, *Bacillus thuringiensis*, *Aspergilus niger* and *Aspergilus oryzae*), yeast (mainly represented by Saccharomyces cerevisiae) and acid lactic bacteria (LAB) (mainly belonging to the species lactobacillus: *Lactiplantibacillus plantarum* subsp. *plantarum*, *Levilactobacillus brevis*, *Lactinocaseibacillus rhamnosus* and *Lactobacillus acidophilus*) [13,14]. Particularly, lactofermentation can be a useful tool for remodeling the polyphenolic composition of vegetables and fruit, enhancing their functional potential [15,16,17].

It is also well known that only some polyphenols occurring in foods are easily bioaccessible at the intestinal level, since a major part of these molecules are bound by various types of interactions to a food matrix, mainly represented by soluble and insoluble fiber and cell wall polysaccharides (PCWs) [18]. The enzymatic activity of microorganisms has been widely documented to be able to split the same types of bound molecules and/or degrade complex polyphenols in smaller ones, which in most cases are more bioaccessible at the intestinal level [13].

In light of the above statements, it can be hypothesized that lactofermentation may play a major role in improving the potential functional features of this fruit. Thus, the aim of the present study was the formulation of lactofermented palm date fruit bars (LDBs) as a potential functional food. In order to reach this aim, we have evaluated (i) the capability of the growth of most diffuse lactobacillus strains in palm date pulp and their survival after 4 weeks of storage at 4 °C, (ii) the effects of lactic acid fermentation on free polyphenolic compounds levels in palm date fruit bars, (iii) the desirable enhancement of LDB antioxidant potential, and (iv) the remodeling of the polyphenolic composition of LDB.

## 2. Materials and Methods

### 2.1. Inoculum and LDB Preparation

*Lactiplantibacillus plantarum* subsp. *plantarum* ATCC14917, *Lactobacillus delbruekii* subsp. *bulgaricus* ATCC 11842, *L. acidophilus* ATCC4356 and *Lactinocaseibacillus rhamnosus* ATCC 7469 (Farmalabor, Canosa, Italy) were reactivated by culturing twice in 25 mL of MRS broth (meat peptone 10.0 gL^−1^; dextrose 20.0 gL^−1^; yeast extract 5.0 gL^−1^; beef extract 10.0 gL^−1^; disodium phosphate 2.0 gL^−1^; sodium acetate 5.0 gL^−1^; ammonium citrate 2.0 gL^−1^; magnesium sulfate 0.1 gL^−1^; manganese sulphate 0.05 gL^−1^; Tween 80 1.0 gL^−1^) (Thermo scientific, Waltham, MA, USA) at 37 °C for 18 h to obtain 10^8^ cells/mL. To prepare the inoculum, bacterial cultures were centrifuged and washed in sterile physiological solution (NaCl 8.5 gL^−1^) and resuspended in 5 mL of the same solution. Fresh palm date fruits were purchased from a local supermarket, were boned, and 200 g of milled date fruit pulp, 100 g of cereals, 18 h cultures (final concentration > 10^6^ CFU/mL) and sterile water were mixed in a food processor mixer for 2 min. For the fermentation, the mixtures obtained were placed in plastic bags and incubated at 37 °C for 48 h. After this time, the pH was measured followed by sample drying up to 15% moisture content. Extrusion was accomplished using a laboratory single screw extruder S-45 (Metalchem Gliwice) after the fermentation time.

### 2.2. Enumeration of Probiotic Microorganisms

The viability of probiotic cultures in the LDBs was determined and expressed as colony forming units (CFU) mL^−1^ on MRS agar (Oxoid, Milan, Italy). Serial dilutions were prepared in sterile physiological solution before being plated onto MRS agar. Plates were incubated at 37 °C for 48 h in an anaerobic system (Oxoid). Probiotic viability was investigated after 48 h of fermentation at 37 °C and after 4 weeks of storage at 4 °C.

### 2.3. LDB Polyphenolic Extraction

In order to evaluate the potential changes in terms of antioxidant power and polyphenolic content, the LDBs were subjected to chemical extraction after the fermentation time and after 4 weeks of storage at 4 °C. LDBs (10 g) were treated with 60 mL of 80% methanol (0.5% formic acid), homogenized for 5 min by ultra-turrax (T25-digital, IKA, StaufenimBreisgau, Berlin, Germany) and shaken on an orbital shaker (Sko-DXL, Argolab, Carpy, Italy) at 300 rpm for 15 min. Then, the samples were placed in an ultrasonic bath for another 10 min, before being centrifuged at 6000 rpm for 10 min. The supernatants were collected and stored in darkness, at 4 °C. The pellets obtained were re-extracted, as described above with 40 mL of the same mixture of solvents. Finally, the extracts obtained were filtered under vacuum, the methanol fraction was eliminated by evaporation, and the water fraction was lyophilized.

### 2.4. HPLC-DAD Analysis

Extracts from the date bars were solubilized with 1% formic acid. Analyses were run on a Jasco Extrema LC-4000 system (Jasco Inc., Easton, MD, USA) provided with a photodiode array detector (DAD). The column selected was a Kinetex^®^ C18 column (250 mm × 4.6 mm, 5 μm; Phenomenex, Torrance, CA, USA). The analyses were performed at a flow rate of 1 mL/min, with solvent A (2% acetic acid) and solvent B (0.5% acetic acid in acetonitrile and water 50:50, *v*/*v*). After a 5 min hold at 10% solvent B, elution was performed according to the following conditions: from 10% (B) to 55% (B) in 50 min and to 95% (B) in 10 min, followed by 5 min of maintenance. Procyanidins, dihydrochalcones, flavanols, and hydroxycinnamic acids were monitored at 280 nm, while flavonols were monitored at 360 nm. For quantitative analysis, standard curves for each polyphenol standard were prepared over a concentration range of 0.1–1.0 μg/μL with six different concentration levels and duplicate injections at each level. The identity of polyphenols was confirmed by comparison of the retention time with the external standard.

### 2.5. Total Phenol Content (TPC)

The total phenol content (TPC) was determined through Folin–Ciocalteau’s method, using gallic acid as standard (Sigma-Aldrich, St. Louis, MO, USA). In brief, 0.1 mL of samples (properly diluted with water in order to obtain an absorbance value within the linear range of the spectrophotometer) underwent an addition of: 0.5 mL of Folin–Ciocalteau’s (Sigma-Aldrich, St. Louis, MO, USA) reagent and 0.2 mL of an aqueous solution of Na_2_CO_3_ 7% (*w*/*v* %), bringing the final volume to 10 mL with water. After mixing, the samples were kept in the dark for 90 min. After the reaction period, the absorbance was measured at 760 nm. Each sample was analyzed in triplicate and the concentration of total polyphenols was calculated in terms of gallic acid equivalents (GAE) [19].

### 2.6. DPPH Assay

The antioxidant activity of the samples was measured with for the radical scavenging ability of the antioxidants present in the sample using the stable radical 2,2-diphenyl-1-picrylhydrazyl (DPPH) (Sigma-Aldrich St. Louis, MO, USA). The analysis was performed by adding 100 µL of each sample to 1000 µL of a methanol solution of DPPH (153 mmol L^−1^). The decrease in absorbance was determined with a UV–visible spectrophotometer (Beckman, Los Angeles, CA, USA). The absorbance of DPPH radical without antioxidant, i.e., the control, was measured as the basis. All determinations were in triplicate. Inhibition was calculated according to the formula: [(Ai − Af)/Ac] × 100 [19].

### 2.7. Statistics

Unless otherwise stated, all the experimental results were expressed as the mean ± standard deviation (SD) of three determinations. Statistical analysis of data was performed by the Student’s *t*-test. *p* values less than 0.001 were regarded as significant.

## 3. Results

### 3.1. Microbial Analysis

The four acid lactic bacteria strains used were capable of growth in palm date bars media without external prebiotic supplementation or pH adjustment, reaching a concentration ranging from 3.1 to 4.9 × 10^9^ CFU/g after 48 h of fermentation (Table 1). The shelf-life analysis performed, consisting of 4 weeks of storage at 4 °C, indicated that the surviving cell was easily affected by the storage, and the fermented product keeps functional probiotic concentration (approximately 10^9^ CFU/g) able to exert beneficial effects in the consumers. Regarding pH, it decreased approximately by a single unit (with a slight difference depending on the bacteria strain used) and remained almost unchanged after the storage time.

### 3.2. Total Polyphenols and Antioxidant Activity

In order to obtain an overview of the effects of lactofermentation on palm date bars’ polyphenolic composition, Folin–Ciocalteau’s assay was performed on hydroalcoholic LDB and control (unfermented bar) extracts. With this assay, we evaluated the non-specific quantitative variation of polyphenolic compounds in hydroalcoholic extracts, thus the change in terms of extractable polyphenols. Un-inoculated date bars exhibited a total phenol content of 44.87 mg GAE (gallic acid equivalent) for date bars. This value increased after 48 h of fermentation with acid lactic bacteria and its positive variation, showing changes in a strain-specific manner. As reported in Table 2, *L. rhamnosus* fermented bars exhibited an increase in total polyphenolic content of 71.58% compared to the control, while the inoculation with *L. bulgaricus* and *L. acidophilus* brought slightly lower results with an increase of 41.19% and 59.03%, respectively. *L. plantarum* fermentation, instead, has shown a completely different influence on the phenolic composition, with a negligible activity on free polyphenols increase. The same trend was also maintained after four weeks of storage at 4 °C, where the relevant increases in free phenolic compounds obtained were preserved with a slight reduction (Table 2). The antioxidant activity of the LDBs and the control, instead, was evaluated by a DPPH test and the results obtained were expressed as mg of trolox equivalent/LDB (Table 3). The results obtained show that the hydroalcoholic extract of *L. rhamnosus* LDB possesses a stronger antioxidant activity than the other fermented versions, with an increase in radical scavenging activity by 39.97%. The other bacteria strains also showed a positive influence on the antioxidant potential of this product. Particularly, *L. acidophilus* fermentation shows a remarkable effect on the antioxidant activity of the present product, with an increase in antioxidant activity of 30.91%, while *L. plantarum* and *L. bulgaricus* LDBs show a weak change in terms of radical scavenging activity, with a slight increase (calculated against the control according to the formula: (TPC fermented products − TPC of control)/TPC control × 100) in TE/LDB of 2.8 to 23.61%, respectively. Additionally, in this case, the storage for 4 weeks at 4 °C seems to not significantly affect the antioxidant potential of the LDBs.

### 3.3. Polyphenolic Composition of LDB

HPLC-DAD quantitative analysis results of the main representative palm date polyphenols occurring in LDBs and the control are reported in Table 4. Data show that fermentation has remarkable effects on the qualitative composition of the date polyphenolic fraction. The total phenolic acid concentrations are increased after the lactic fermentation. The gallic acid amount is significantly increased (8-fold compared to the control) in *L. rhamnosus* LDB. Lower results, but still significant, were obtained by fermentation with the other strains (Table 4). The amount of caffeic acid, ferulic acid, and syringic acid are almost doubled in the fermented product and specifically, the highest increase was achieved, once again, in *L. rhamnosus* LDB. Concerning the phenolic acids, only the chlorogenic acid concentration drastically decreased after lactofermentation; indeed, its concentration halved in formulated products. In regard to the flavonoid composition, interestingly, the amount of quercetin is increased around +50% in all the different versions of LDB formulations. However, the latter results are accompanied with a relevant decrease in quercitrin and isoquercetin levels.

## 4. Discussion

Our results indicated that all the inoculated bacteria strains in date fruit pulp are capable of growing well, without nutrient supplementation or pH alteration. These findings agree with previous works, which have proposed different vegetable or fruit juices as good media for probiotic growth [13,20,21]. During the last decade, the consumption of probiotic products is a trend in continuous evolution due to its widely documented health-promoting effects [21,22,23,24,25].

It is fundamental to emphasize that, to exert beneficial effects in the host, probiotic bacteria must be alive and abundant in the product at the time of consumption [20]. Today, no general agreement has been established regarding the minimum concentration of probiotics necessary to obtain healthy effects in humans; however, a daily intake ranging from 10^6^ to 10^8^ CFUmL^−1^ is generally recommended [26]. LDB products possessed, after fermentation, a probiotic concentration, largely above the minimum daily probiotic intake recommended. Particularly, *L. rhamnosus* strain showed the best growth in date fruit pulp, using the date fruit components as a nutritional source for their growth and development, reaching the concentration of 4.9 × 10^9^ CFU/g of LDB. Our results are in line with findings of other authors, that indicated the *L. rhamnosus* strain as the strain most able to survive in unfavorable conditions such as fruit or vegetable juice [20,21]. The latter probiotic potential of the formulated products agrees with another relevant nutraceutical feature of this one: the increase in bioaccessible polyphenolic fraction. The results obtained after the lactic fermentation of palm date pulp indicated a general increase in total polyphenolic level (Table 2) and the related antiradical activity of fermented products (Table 3).

These positive chemical and biological changes vary in a strain-specific manner (Table 2 and Table 3), *L. rhamnosus* has shown to be the most effective stain able to improve the biochemical characteristics of the LDBs. These data would be related to the bacteria enzymatic activities (hydrolase, esterase, etc.), that would catalyze the release of phenolic compounds from soluble and insoluble date fruit fiber.

Consequently, the intestinal date polyphenols’ bioaccessibility and consequential potential bioavailability may increase [13,27,28,29]. It is widely documented that the polyphenolic compounds are massively absorbed in the small intestine, and it is also well known that the intestinal bioaivability of the polyphenols is low, especially due to their high molecular weight and polarity [30]. In this scenario, lactofermentation is a precious strategy to release polyphenolic compounds from the food matrix, but also a tool to remodel the polyphenolic composition of palm date fruit in favor of a smaller and less polar one, thus reasonably creating more absorbable bioactive molecules. HPLC-DAD analysis of the LDB polyphenolic extracts indicated a drastic change in terms of polyphenolic composition. The fermented version of palm date bars showed a radical reduction in chlorogenic acid in favor of its molecular component: caffeic acid [31]. This molecule may exert more health-promoting effects than its precursor and, due to its lower polarity and dimension, it is better absorbed in the small intestine than its precursors [32]. The observed increase in cinnamic acids (i.e., ferulic and caffeic acids) is also related to feruloyl esterase enzymes (E.C. 3.1.1.73), produced by bacteria, responsible for catalyzing the resolution of polyphenolic esterified forms to the vegetable cell wall, that turn into the release of free phenolic acids, available to be absorbed at the intestinal level [33]. Furthermore, the release of gallic acid in LDB was observed. This compound may be released via tannase activity. Tannase or tannin acyl hydrolase (EC 3.1.1.20) catalyzes the hydrolysis of ester bonds that occur in hydrolysable tannins and gallic acid esters, and releases glucose and gallic acid [34]. The fermented version, moreover, was also enriched in quercetin, a not polar polyphenol largely absorbed at the intestinal level. Microbial α-rhamnoside (widely produced by *L. rhamnosus*) can hydrolyze quercitrin (quercetin 3-O- rhamnoside) in its aglycone form (quercetin). Similarly, rutin may be hydrolyzed by a-rhamnosidase to produce quercetin-3-O-glucoside, and then further hydrolyzed by α-glucosidase (bacteria enzyme) to release quercetin [35,36]. In light of these considerations, the polyphenolic richness of these products was not only fortified by the deep remodeling operated by bacteria activities, but the general polyphenolic stability was improved by pH change (from neutral to mild acid), enhancing the intestinal bioaccessibility and bioavailability of these bioactive compounds [37].

## 5. Conclusions

On the basis of functional ingredients occurring in date fruit, LDB products, and especially *L. rhamnosus* LDB, may be proposed as a prototype of functional food, mainly indicated for athletics nutrition and supplementation. LDB products may be considered as bi-functional, because after the biotransformation, LDB increases the rate of free and simple phenolic compounds, which are more absorbable at the intestinal level, and, at the same time, acts as a carrier of probiotics. The higher amounts of free phenolic compounds may be a precious support able to contrast the strong oxidative stress to which athletes are constantly subjected. Moreover, the high glucose content is a good source of energy, and the high mineral levels may rebalance the loss of minerals during sportive activities. Finally, the active probiotics may aid athletes with secondary health benefits that could positively influence physical performance through improved recovery from fatigue, enhanced immune function, and the maintenance of healthy gastrointestinal and upper respiratory tract function [38]. Accordingly, a recent clinical trial has proven the importance of probiotic supplementation to contrast the high oxidative stress level [39] and to increase muscle strength and resistance [40]. Undoubtedly, further in vivo studies are necessary to confirm such promising results.

## Figures and Tables

**Table 1:** Monitoring of pH and cell survival (expressed as CFU/ g of LDB) in fermented and unfermented version of palm date bars. Means and standard deviations for *n* = 3.

Time	Surviving Cells (CFU/g)			
	*L. acidophilus*	*L. bulgaricus*	*L. plantarum*	*L. rhamnosus*
0	1.4 ± 0.2 × 10^6^	1.2 ± 0.3 × 10^6^	1.6 ± 0.4 × 10^6^	1.3 ± 0.2 × 10^6^
48 h	4.2 ± 0.3 × 10^9^	3.1± 0.4 × 10^9^	4.4 ± 0.2 × 10^9^	4.9 ± 0.3 × 10^9^
4 weeks	2.8 ± 0.2 × 10^9^	1.9 ± 0.2 × 10^9^	2.1 ± 0.3 × 10^9^	2.8 ± 0.2 × 10^9^
	**pH**			
	*L. acidophilus*	*L. bulgaricus*	*L. plantarum*	*L. rhamnosus*
0	4.90 ± 0.01	4.97 ± 0.2	4.82 ± 0.20	4.84 ± 0.08
48 h	3.20 ± 0.09	3.53 ± 0.30	3.47 ± 0.07	3.21 ± 0.09
4 weeks	3.17 ± 0.02	3.22 ± 0.36	3.23 ± 0.12	3.14 ± 0.09

**Table 2:** Total phenol content (TPC) evaluated by Folin–Ciocalteu method in fermented and unfermented date bars after 48 h of fermentation at 37 °C and 4 weeks of storage at 4 °C. Data are expressed as mean value (mg gallic acid equivalents (GAE)/g LDB) ± SD of three repetitions.

mg GAE/Date Bars				
Probiotic Strains	48 h of Fermentation	4 Weeks at 4 °C	% Increase in Free Polyphenols after 48 h of Incubation	% Increase in Free Polyphenols after 4 Weeks at 4 °C
*L. acidophilus*	71.34 ± 0.08 *	63.13 ± 0.32 *	59.03	45.82
*L. bulgaricus*	63.29 ± 0.16 *	59. 67 ± 0.20 *	41.19	36.12
*L. plantarum*	47.27 ± 0.04 **	49.94 ± 0.05 **	4.91	7.02
*L. rhamnosus*	77.56 ± 0.15 *	71.34 ± 0.61 *	71.58	61.54
Control	44.87 ± 0.07	43.88 ± 0.10		

Statistical significance is calculated by Student’s t-test analysis: * *p* < 0.0001 TPC of *L. acidophilus*, *L. bulgaricus*, *L. rhamnosus* LDB vs. control (44.87 mg of GAE/date bars) for 48 h fermented sample; ** *p* < 0.001 TPC of *L. plantarum* LDB vs. control (44.87 mg of GAE/date bars) for 48 h fermented sample; * *p* < 0.0001 TPC of *L. acidophilus*, *L. bulgaricus*, *L. rhamnosus* LDB vs. control (43.88 mg of GAE/date bars) for 4-week fermented sample; ** *p* < 0.001 TPC of *L. plantarum* LDB vs. control (43.88 mg of GAE/date bars) for 4-week fermented sample.

**Table 3:** Radical scavenging activity evaluated by DPPH method in fermented and unfermented date bars after 48 h of fermentation at 37 °C and 4 weeks of storage at 4 °C. Data are expressed as mean value (mg Trolox equivalents (TE)/LDB) ± SD of three repetitions.

mg TE/Date Bars				
Probiotic Strains	48 h of Fermentation	4 Weeks at 4 °C	% Increase in Antioxidant Activity 48 h of Incubation	% Increase in Antioxidant Activity after 4 Weeks at 4 °C
*L. acidophilus*	140.64 ± 0.31 *	111.78 ± 0.79 *	30.91	21.74
*L. bulgaricus*	133.53 ± 0.91 *	128.83 ± 0.15 *	23.61	40.00
*L. plantarum*	111.05 ± 0.13 **	108.33 ± 0.58 **	2.80	17.73
*L. rhamnosus*	150.13 ± 0.15 *	141.26 ± 0.43 *	39.97	53.50
Control	108.03 ± 0.16	92.02 ± 0.17		

Statistical significance is calculated by Student’s t-test analysis: * *p* < 0.0001 TPC of *L. acidophilus*, *L. bulgaricus*, *L. rhamnosus* LDB vs. control (108 mg of TE/date bars) for 48 h fermented sample; ** *p* < 0.001 TPC of *L. plantarum* LDB vs. control (108.03 mg of TE/date bars) for 48 h fermented sample; * *p* < 0.0001 TPC of *L. acidophilus*, *L. bulgaricus*, *L. rhamnosus* LDB vs. control (92.02 mg of TE/date bars) for 4-week fermented sample; ** *p* < 0.001 TPC of *L. plantarum* LDB vs. control (92.02 mg of TE/date bars) for 4-week fermented sample.

**Table 4:** Polyphenolic composition of date bars (control) and of its lactofermented versions (LDB) formulated by fermentation with different *Lactobacillus* strains.

Phenolic Compound	*L. acidophilus* LDB	*L. bulgaricus* LDB	*L. plantarum* LDB	*L. rhamnosus* LDB	Control
Gallic acid	1.87 ± 0.07 *	1.74 ± 0.09 *	1.39 ± 0.07 **	8.05 ± 0.03 *	1.40 ± 0.03
Syringic acid	3.97 ± 0.02 *	3.77 ± 0.03 *	3.53 ± 0.03 **	4.15 ± 0.02 *	2.46 ± 0.03
Caffeic acid	3.97 ± 0.03 *	3.73 ± 0.02 *	3.41 ± 0.03 **	4.42 ± 0.03 *	3.32 ± 0.03
Ferulic acid	5.17 ± 0.04 *	4.88 ± 0.02 *	4.73 ± 0.03 **	5.46 ± 0.03 *	4.26 ± 0.04
Chlorogenic acid	2.82 ± 0.03 *	3.01 ± 0.02 *	3.93 ± 0.03 **	2.64 ± 0.03 *	4.73 ± 0.05
Cathechin	1.56 ± 0.03 *	1.55 ± 0.05 *	1.43 ± 0.03 **	1.78 ± 0.03 *	1.34 ± 0.02
Rutin	0.56 ± 0.04 *	0.48 ± 0.01 *	0.56 ± 0.06 **	0.42 ± 0.03 *	0.91 ± 0.02
Quercitrin (Quercetin 3-O-rhamnoside)	0.23 ± 0.02 *	0.26 ± 0.02 *	0.21 ± 0.03 **	0.29 ± 0.02 *	0.78 ± 0.02
Isoquercetin	0.28 ± 0.02 *	0.26 ± 0.03 *	0.23 ± 0.03 **	0.1 ± 0.03 *	0.76 ± 0.03
Quercetin	0.21 ± 0.01 *	0.29 ± 0.01 *	0.25 ± 0.03 **	0.32 ± 0.01 *	0.11 ± 0.05

Statistical significance is calculated by Student’s t-test analysis: * *p* < 0.0001 polyphenolic composition of *L. acidophilus*, *L. bulgaricus*, *L. rhamnosus* LDB vs. control; ** *p* < 0.001 polyphenolic composition of *L. plantarum* LDB vs. control.

## Data Availability

The data used to support the findings of this study are included within the article.
